# The toolbox for mosquito vector research

**DOI:** 10.1186/s13071-025-07008-2

**Published:** 2025-09-24

**Authors:** Sebastian Duran-Ahumada, Vivian Petersen, Michael Futo, Mathieu Zamy, Timothy Pereira, Bianca C. Burini

**Affiliations:** 1 Florida Medical Entomology Laboratory, University of Florida, Vero Beach, FL 32962 USA; 2 Department of Entomology, Minnie Belle Heep Center, Texas A & M University, College Station, TX 77843-2475 USA

**Keywords:** Mosquito collection, Pathogen transmission, Vector competence, Molecular identification, Infection studies, Laboratory colonization, Host–pathogen

## Abstract

The work of collecting mosquitoes in the field is essential for improving knowledge about species of known and unknown vector status, their invasion dynamics, and further understanding their involvement in circulating pathogens of medical and veterinary importance. Over the years, different techniques have been developed to capture mosquitoes for entomological, pathogen transmission, and surveillance studies. For entomological and transmission dynamic research to be reliable, it is essential for mosquito specimens to be correctly identified so that their role in pathogen transmission can be appropriately assessed. Currently, modern molecular techniques support traditional morphological taxonomic identification and provide faster and more assertive identification. It is also important to test the susceptibility of potentially relevant mosquitoes or mosquitoes with unknown vector status in the laboratory to identify which species could participate in pathogen transmission. This last step toward fully performing vector incrimination of mosquito species and understanding their interactions with relevant pathogens requires proper handling of live specimens and laboratory colonization under artificial conditions to perform artificial infection studies. In this work, we aim to underscore the significance of the available tools for entomological studies and pathogen transmission research while also offering insights into the principles behind recent technological advancements that enhance the effectiveness and reliability of these studies.

**Graphical Abstract:**

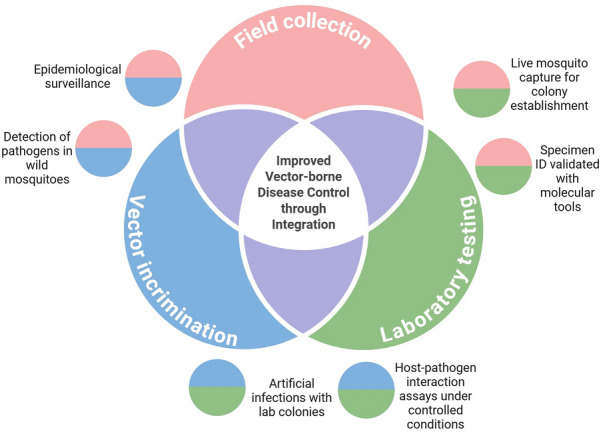

**Supplementary Information:**

The online version contains supplementary material available at 10.1186/s13071-025-07008-2.

## Background

Mosquito surveillance to understand population dynamics, species composition, and distribution is of utmost importance in vector control programs, and it is the basis of informative field epidemiology [1]. Mosquito surveillance is also relevant for detecting invasive species that can transmit pathogens such as viruses, parasitic nematodes, and protozoa, as some mosquito species can act in pathogen–host cycles in newly invaded regions [2]. Currently, factors such as increased human mobility, global trade, and climate change are facilitating the introduction and establishment of mosquito species in regions where they were previously absent [3]. Today, it is estimated that more than 129 countries have environmental and climatic conditions conducive to harboring and developing invasive species of mosquitoes [4]. These invasive species may begin to transmit pathogens in their newly occupied region, so surveillance targeting mosquito species of public health concern and laboratory research into their likelihood to transmit pathogens is essential.

Continuing with this surveillance work, it is necessary to capture mosquitoes in the field, which is aided by an ample set of ever-growing collection techniques that can be chosen according to the objectives set by the study and its context (i.e., features of the life history of the species including host-preference, egg-laying preferences, patterns of activity) [5]. Moreover, accurately identifying field-captured mosquitoes is essential for precisely establishing the epidemiological patterns of the pathogens of interest. Some of the relevant mosquito species for public health are difficult to identify solely by visible morphological characteristics, as many of these species can belong to complexes comprised of cryptic species (i.e., morphologically indistinguishable taxa) that belong to different evolutionary lineages. Mosquito species identification can be further aided by molecular techniques based on differences in the sequences of genetic markers (e.g., *mitochondrial cytochrome c oxidase subunit I* (COI), *nuclear DNA internal transcribed spacer 2* (ITS2), mitochondrial gene coding for *16S ribosomal RNA*, nuclear *white* gene) [6, 7] and other DNA-based identification techniques. In addition, morphometric tools such as geometric wing morphometry can assist in identifying some members of a species complex [8].

**Fig. 1:**
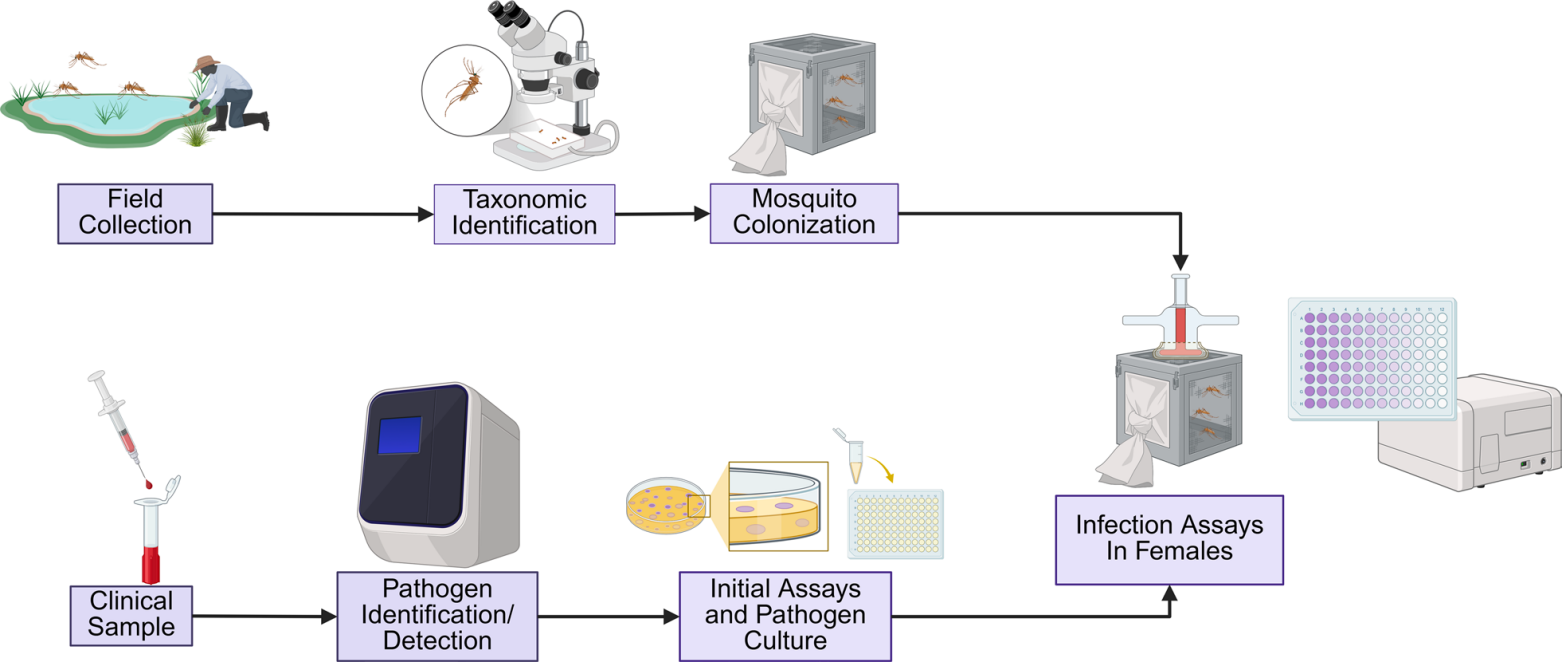
Generalized workflow for mosquito vector research. Created with Biorender

In addition to entomological studies, the importance of research centered on pathogen detection in field-collected specimens stands out. For example, early detection of circulating pathogens can prompt the appropriate implementation of combinations of targeted control measures and initiate vaccination campaigns, when available, to prevent or mitigate disease outbreaks [9]. Furthermore, it is crucial to assess the susceptibility of mosquitoes to different pathogens to better understand their epidemiological impacts and participation in transmission cycles and, ultimately, aid targeted vector control along with other relevant public health prevention and mitigation actions to decrease transmission risk. Studies on mosquito susceptibility and vector incrimination can be approached by detecting and characterizing pathogens from field-collected specimens through various techniques (e.g., tissue culture or polymerase chain reaction [PCR]). Vector incrimination studies are complemented through artificial infection assays with mosquitoes sourced from laboratory-reared stocks at different degrees of establishment (e.g., first or second generations are preferred) and the aid of pathogen isolates derived from clinical tissue samples (e.g., serum or blood) or laboratory-cultured pathogens (e.g., NF54 *Plasmodium falciparum* strain [10]). Figure 1 shows a general workflow outlining both mosquito-centric and pathogen-centric approaches, highlighting key actions commonly undertaken in mosquito vector research. This schematic is a central framework for the topics discussed further.

This review explores how studying field-collected and field-derived mosquitoes and their interactions with mosquito-borne pathogens can advance medical entomology and the epidemiological study of mosquito-borne diseases and control. We highlight traditional approaches and recent innovations in the study of mosquito biology, ecology, and host–pathogen interactions, emphasizing their practical applications in improving mosquito vector research, disease surveillance, and control strategies.

### Mosquito collection

Effective mosquito collection is vital for vector surveillance and research. Mosquitoes can be captured as either adults (males, females) or in their immature forms (eggs, larvae, pupae) through different collection methods, each with advantages and restrictions. These methods can be broadly classified into passive and active collections. Passive collection relies on traps and/or baits to attract specimens, while active collection involves directly searching for them in their habitats using tools such as portable aspirators, sieves, dippers, or handheld nets. The selection of collection methods, devices, environment, and sampling design must be driven by the biology and ecology of the species of interest, the specific life stage, the physiological state, and the goal of the study [5].

On the active collection side, entomological aspirators can provide large numbers of adult forms of both sexes for the analysis of their vector competence in the laboratory [11] and also aid in detecting transovarial pathogen transmission [12]. However, a good example of passive mosquito collection is offering artificial shelters, which can prove helpful for collecting engorged females [5, 13–15]. To capture engorged and host-seeking female mosquitoes in host preference studies, Centers for Disease Control and Prevention (CDC) light traps equipped with a CO_2_ attractant can be used [16]. Furthermore, immature and adult mosquito collection methods can help inform targeted vector-borne disease control and insecticide resistance testing. Below are overviews of the collection methods of adult and immature forms, along with discussions of their functioning and use.

#### Adult collection

Several active and passive adult mosquito collection methods have been developed [5, 17–23]. Hereby, some of the most relevant, widely used, recommended traditional, and recent adult collection methods are presented and discussed [5, 24–27].

##### BG-Sentinel trap

The BG-Sentinel trap (BioGents, Rosenburg, Germany) is widely regarded as the gold standard for capturing *Aedes aegypti* and *Aedes albopictus* [23, 28–30]. It has also been reported to collect other species of the *Stegomya* subgenus that can act as secondary dengue virus (DENV) vectors, such as *Aedes polynesiensis* [31]. This trap effectively captures host-seeking females by mimicking convection currents produced by a human host, along with visual cues [24, 25] that drive host-seeking mosquitoes to its top funnel intake, where they are pulled toward a collection bag (Additional File 1: Supplementary Fig. S1A). Its efficiency is further enhanced by using attractive lures such as carbon dioxide, octanol, and BG-Lure, which is a chemical attractant formulation replicating human skin odors [24, 25].

Another iteration of this trap, the BG-Sentinel 2, was evaluated for capturing *Ae. albopictus* in southern France [32]. This study showed that the capture rates significantly increased with the addition of carbon dioxide, and that the BG-Lure was effective by itself, with a combination of *R*-1-octen-3-ol and carbon dioxide being the best for capturing males. In contrast, only the BG-Lure and carbon dioxide effectively attracted females. Interestingly, an accessory (BG-Counter, Biogents) for this trap that offers remote monitoring with automated mosquito counting has been developed and is currently available [33].

Initially intended to replace the human landing catch in the collection of *Ae. aegypti* [25], the use of the BG-Sentinel 2 has been extended to catching other mosquito species, such as *Ae. albopictus*, with similar performance as the BG-Sentinel 1 [23] and of particular interest for outdoor collection. Its limitations in outdoor environments, such as exposure to rain and potential sample damage, can be effectively mitigated by using simple protective measures. These include manufacturer-provided covers, deploying the setup under portable camping tables, or selecting naturally sheltered locations.[34]. A field experiment assessing another version of this trap, called the BG-Malaria trap, tailored for collecting *Anopheles* mosquitoes, tested different alternatives to the base design and material of the collection bag, given that the original materials caused damage to collected mosquitoes through the loss of their hind legs, with success [35].

A limitation of the initial versions of the BG-Sentinel trap was the absence of a light source, which could otherwise enable the collection of a broader range of nocturnal mosquito species and enhance collection rates. The modular version, the BG-Pro, offers the option to add ultraviolet (UV) light-emitting diode (LED) sources and allows reconfigurations to emulate other adult mosquito traps for surveillance and control purposes [36]. Additional field research has shown an increase in female *Anopheles gambiae* s.l. [37] and *Ae. albopictus* [38] collected by traps with heat sources, suggesting that adding different attractants can increase the capture of different species.

##### CDC light trap

Introduced to the world in 1962 [39], the CDC light trap (Additional File 1: Supplementary Fig. S1B), along with its miniature version, is a widely used tool for mosquito surveillance that primarily relies on light as an attractant but can be paired with carbon dioxide to improve collection efficiency [40, 41]. The CDC light trap is a suction trap that collects mostly unfed female mosquitoes in a container that can be a mesh cone or designed for the goals of any given study [5, 17]. This trap typically uses an incandescent CM-47 bulb that emits a broad wavelength, including visible (color temperature 2256 K, 7 lumens) and some infrared light, but this can be replaced with an LED to optimize mosquito capture [42], making the CDC light trap more generalist than the BG-Sentinel trap. It is crucial to note that the invention of the CDC light trap was driven by the need for a more field-friendly alternative to the New Jersey Light Trap (NJLT) [5, 17]. The NJLT, a widely used mosquito surveillance tool, consists of a steel or brass-built structure equipped with a light source and a fan-powered suction system to attract and capture mosquitoes [43, 44]. However, its heavy and bulky design made field deployment challenging. This limitation inspired the development of the CDC light trap, which offers a more lightweight, portable, and easily deployable solution while maintaining effective mosquito collection capabilities.

Other traps with similar operating principles as the CDC light, such as the Blackhole mosquito trap, have been shown to increase the nighttime collection of *Anopheles* spp. by employing different light sources such as UV LED and UV fluorescent lights [45]. Nevertheless, different colored lights showed no advantage over regular incandescent lighting in sampling *Aedes* spp. vectors of Rift Valley Fever virus in hotspots of Kenya with CDC light traps [46].

Modifying attraction cues relevant to their functioning can improve collection rates and species representation of CDC light trap-based collections. This way, carbon dioxide has become commonplace in their deployment. The use of CO_2_ was initially explored in Vietnam back in 1972 using dry ice [47]. In a study conducted in northwestern Thailand, non-baited CDC traps were compared with carbon dioxide-baited traps, resulting in a significantly increased capture of *Anopheles* spp. and *Culex* spp. mosquitoes due to the addition of CO_2_ [48]. The results also indicated that *Anopheles minimus* was more frequently trapped indoors, while *Culex vishnui* was more abundant in outdoor traps. Seasonal variation influenced capture rates, with the highest numbers observed during the hot season. These findings highlight the importance of optimizing mosquito surveillance strategies by incorporating carbon dioxide baiting to enhance trap effectiveness. They also considered that seasonal variations can significantly influence mosquito population density [48]. However, obtaining and transporting dry ice or other CO_2_ sources in remote areas can be challenging. To address this, various alternative methods have been explored with these and other traps, including CO_2_ generation through sugar fermentation, inorganic chemical reactions, and fossil fuel combustion [49–51].

A notable limitation of this trap is the fan at the top of the collection bag, which sometimes results in mechanical damage to specimens, affecting their correct identification and making it more challenging to use this trap for colonization efforts. This way, a study comparing the CDC light trap with a similar trap, differing only in fan position and lacking a light source, showed that *Anopheles farauti* had enhanced survival despite the trap capturing fewer mosquitoes [52]. Additional improvement considerations include a pull system to minimize the physical damage of mosquitoes and increase their survival for subsequent goals, including colony establishment or obtention of F1 individuals for complementary work.

Notably, other CDC light traps and traps inspired by their principles have been developed with specific goals in mind. Interestingly, it has been hypothesized that this trap could miss out on mosquito species that swarm around their host and would be unlikely to be attracted by the trap cues. Hence, a set of smart solutions comprising the addition of motion sensors to activate the trap coupled to these modified traps by an offered animal feeder, meant to attract wildlife, have shown to be likely to circumvent such potential exclusion biases in mosquito sampling [53]. These traps are highly versatile and can be installed at various heights, increasing the likelihood of capturing mosquitoes from different strata. However, studies have shown that they effectively collect large numbers of West Nile virus vector species, regardless of their deployment height [54].

##### Shannon trap

The Shannon trap is a ground-based mosquito trap consisting of a white fabric enclosure (1.3 m × 3.0 m × 2.0 m) with two lateral flaps (0.6 m × 3.0 m × 1.0 m). It is effective for mosquito diversity studies, particularly for nocturnally active species. It uses carbon dioxide, a strong attractant for daytime captures, while its internal light is crucial for attracting mosquitoes during nighttime collections [5]. Mosquitoes drawn to the trap can be collected using entomological aspirators or death tubes. A limitation of this method is the need for human maintenance to collect specimens, which carries a risk of pathogen exposure from mosquito contact. This exposure risk is compounded by the fact that Shannon traps can bias the sampling of mosquitoes such *Anopheles* spp. with anthropophilic tendencies [55]. Interestingly, simple modifications of this trap, such as the use of black fabric, have shown notable increases in capturing non-mosquito vectors of arthropod-borne diseases such as sandflies [56].

##### Human landing catch

One passive collection method is the human landing catch (HLC), in which mosquitoes are aspirated by collectors with their lower legs exposed to lure mosquitoes coming to feed on them [17]. HLC can be very effective for mosquito capture, characterization of biting patterns, and colony establishment. However, there are some ethical concerns [55, 57–59]. This is because, in the “human bait” technique, the collector may fail to capture the mosquito before it takes a blood meal, increasing the risk of pathogen transmission [55]. We believe that the health of the professional is a priority, and therefore, techniques that involve direct contact with the vector should be avoided. Infection likelihood can be mitigated in certain instances, provided risk is correctly assessed and managed [60]. One such approach is the provision of chemoprophylaxis [61] during HLC activities, which has been shown to significantly reduce malaria infection rates among collectors compared with non-collectors [60]. Alternative passive trapping tents have been developed to reduce the risks associated with HLC. These traps take advantage of the natural attraction of exophagic (primarily feeding outdoors) and endophagic (primarily feeding indoors) *Anopheles* spp. mosquitoes to human hosts, providing a safer and more effective collection method. [62–65]. Nevertheless, the safety of these human-baited options needs to be correctly assessed before considering any wide implementation.

##### Aspirator-based adult mosquito collection

Aspirators provide an efficient method for collecting resting mosquitoes indoors and outdoors (Additional File 1: Supplementary Fig. S1C). Two widely used models are the CDC Backpack Aspirator, which is particularly effective for capturing mosquitoes from indoor resting sites [66], and the Prokopack Aspirator, a lighter and more affordable alternative that enhances accessibility for mosquito collection, especially in resource-limited settings [11]. In addition, electric entomological aspirators, constructed from polyvinyl chloride (PVC) or tarpaulin tubes with a motorized suction system, are used to capture mosquitoes in flight or resting in vegetation and houses. These devices come in various sizes depending on study objectives [67, 68] and are valuable for host preference research and pathogen surveillance, as they enable the collection of resting engorged females. They can also complement mosquito diversity in collections with specific goals. For example, in Florida, a combination of pop-up resting shelters and a large-diameter aspirator proved optimal for collecting key vectors of Venezuelan equine encephalitis [69]. The results also revealed that while certain traps effectively capture engorged females of some species, aspiration is more efficient for collecting engorged females of other species. Therefore, active and passive sampling methods can complement each other for more comprehensive mosquito collection [69]. However, some aspirators have proven reliable tools for quantifying indoor mosquito populations, mainly using standardized sampling periods and strategies. They also play a crucial role in evaluating the effectiveness of vector control interventions, measuring natural infection rates with arboviruses, and supporting targeted sampling to assess transovarial transmission of arboviruses [11, 12, 70–72]. Nevertheless, in terms of active mosquito collection, considering classic collection methods such as hand nets could aid in increasing mosquito species representation in studies dealing with mosquito biodiversity and linking species to pathogen transmission [73].

#### Collection methods for gravid females and immature forms

##### Trapping of gravid females and sampling of eggs

Oviposition traps attract gravid females using water and chemical attractants. These traps draw females seeking to lay their eggs into provided containers, where eggs can be collected either from the water, the container itself, or a provided substrate (Fig. 1D). Some traps, such as the Biogents *Aedes* gravid trap, the CDC autocidal gravid ovitrap, and the CDC gravid trap, also allow for egg development, enabling later larval collection, while others feature autocidal or entrapping mechanisms to capture the females themselves [21, 74]. The eggs and larvae can be collected for identification and population monitoring [75–77] and used to establish laboratory colonies. While traps for gravid females are not typically efficient for wide species representation in adult mosquito collection [30], they can be very effective in sampling blood-fed females to detect arboviruses through reverse transcription– quantitative polymerase chain reaction (RT–qPCR) [78].

The range of species attracted by passive trapping for egg sampling can be influenced by several key factors, such as trap construction (e.g., design, substrate features) and the combination of attractants used (e.g., vegetable material infusions, microbiota, or semiochemicals such as geosmin). As a result, the array of egg-capture devices is diverse, spanning from commonly used ovitraps for trapping *Aedes* spp., originally designed for detecting *Ae. aegypti*, consisting of simple dark containers with a substrate and water, sometimes with vegetable infusions, to more specialized setups such as artificial pools and ponds equipped with complex mechanisms for retrieving *Anopheles* spp. eggs [5, 75].

Similarly, the above factors can drive the array of species, attraction, and effectiveness in trapping gravid female mosquitoes of different species toward traps mimicking suitable breeding sites/containers. Furthermore, gravid females can be retrieved dead or alive depending on the operating principles of the selected trap. The Reiter trap is an excellent example of revamping the components of already existing traps. This trap repurposed the fan used in the CDC light trap to collect females attracted to a container providing water with an attracting hay infusion initially meant to collect *Culex* spp. [79]. Interestingly, this trap has been widely used in mosquito surveillance despite its tendency to damage specimens [5]. Nevertheless, a modified version of the Reiter trap, the Reiter–Cummings trap, is a redesign that minimizes specimen damage and that has been widely adopted by mosquito surveillance programs [5, 80]. It has since been revisited and redesigned to collect live mosquitoes using a novel, modifiable design. This updated trap emulates the original and other trap types without forcing specimens through a fan, thereby minimizing mechanical damage [81].

Traps designed primarily to collect gravid *Aedes* spp. mosquitoes are common and even commercially available. This is made possible by the preference of certain *Aedes* species for breeding in man-made containers. Modern commercially available traps, such as the Biogents Gravid *Aedes* trap (BG-GAT) and Autocidal Gravid Ovitraps (designed by the CDC), use a water volume with a grass or hay infusion as an attractant. Once a gravid female mosquito enters the trap, it is funneled into a chamber separate from the water surface. In this chamber, the mosquito is immobilized (e.g., with canola oil or sticky sheets) or killed/knocked down (e.g., by a piece of long-lasting insecticidal-treated bed net or insecticide-coated surfaces). A great example of a trap designed to collect live *Aedes* spp. and *Ochlerotatus* spp. females are provided by the smart design of the female-retaining *Aedes* ovitrap built by Landry and DeFoliart to assess age through age-grading by changes in ovariole dilatations [5, 82].

Trapping *Anopheles* spp. is difficult given their preference for mostly natural or seminatural breeding sites. Nevertheless, simple contraptions such as floating sticky acetate sheets have been used to capture *An. gambiae* s.l. in Tanzania [83] and *An. gambiae* s.s. in Kenya [84]. Electric nets surrounding breeding containers, combined with sticky surfaces, were also attempted for capturing *An. gambiae* s.s. in Kenya with promising results [84]. Another electricity-powered trap has been developed and tested for the collection of gravid *An. gambiae* s.s. The OviART trap comprises a water-filled bucket sunken and flush with the ground, with the opening of a fan-powered suction tube aligned with the water surface. This trap showed increased collection by 60% (relative risk [RR] 1.6, 95% confidence interval [CI] 1.2–2.2; *P* = 0.001) when compared with the electric net/sticky trap configuration previously described [85]. It is noteworthy that the OviART trap was expressly designed to collect live specimens and that in further trials exceeded the trapping performance of other alternatives [86]. Interestingly, BG-Sentinel traps modified to perform as gravid mosquito traps (buried in the ground and filled with water and different attractants) have been tested to analyze the chemical attraction of *An. gambia* s.s. [87] and to test the effect of rice volatiles in *Anopheles arabiensis* [88].

Direct active collection of eggs can be complex for certain species of mosquitoes, especially those choosing natural breeding sites (e.g., difficulty in accessing or in finding eggs); it can also lead to the underrepresentation of cryptic breeding sites that are hard to locate [5]. However, direct active collection of eggs of species that exploit diverse man-made containers, such as *Aedes* spp., with the aid of repurposing simple kitchen utensils is possible [5]. More uses of adapted household utensils and complex, specialized contraptions have been recorded for the active and passive collection of mosquito eggs of different mosquito species occupying natural breeding sites [5].

##### Active larvae and pupae sampling

Larvae and pupae of some mosquito species can be collected by inspecting water-holding containers near human homes or water bodies in the wild. These breeding sites can be investigated using sieves, dippers, and various devices designed for collecting, sifting, dredging, or skimming water or substrates. Such tools may include modifications or attachments tailored to specific environments, especially those with vegetation or debris that can conceal immature mosquito stages when disturbed [5]. In the case of DENV vectors, larval and pupal collection-derived metrics can have practical uses and help with estimating the population density of adults and thus gauge transmission risk. However, these metrics lack sensitivity because of the difficulty in identifying all breeding sites and reliance on homeowners’/landowners’ consent [89].

##### Mosquito identification

With 3729 extant species, classified into two subfamilies and 110 genera, the Culicidae family is abundant, with many species responsible for disease transmission [90]. After mosquitoes are collected, the correct identification of species is an essential part of surveillance and a determining factor for effective control strategies. Mosquito species complexes can be morphologically indistinguishable but are genetically different, with distinct ecology and host preferences. The failure to correctly identify vector species can lead to the wrong administration of vector control strategies in regions where they are most needed, particularly in areas with limited resources [91]. Unequivocal species identification will unarguably decrease the likelihood of wrong species vector incrimination and make targeted control efforts more effective.

Traditionally, mosquito identification is based on external morphological differences and identification keys. Although visual-based methods offer low cost and complexity advantages, caution should be taken. The presence of unknown species complexes or complexes of morphologically similar species can make identification difficult, and trained experts are required to make proper distinctions between morphologically similar structures [92]. In addition, the accurate distinction between morphological characters can be compromised owing to human error or deemed impossible if the specimen is damaged owing to poor handling, trapping conditions, or inadequate preservation or mounting.

Another identification method is wing geometric morphometrics, a relatively simple method to adopt owing to affordable computational and software needs, and the digitalizing applications do not require extensive training [93, 94]. It uses the intersections of mosquito veins as landmarks and analyzes the variability of distances between points from different samples. This has been broadly used for intraspecific variation and interspecific discrimination, among other applications (reviewed in [95]). This technique has been successfully used to investigate the population variability of neglected mosquitoes such as *Ochlerotatus scapularis* [96], morphological polymorphism of *Aedes scapularis* [97], differentiation between the almost identical *Culex pipiens* and *Culex torrentium* [98], and differentiation of sympatric species of *Anopheles* [99]. Visual-based methods or wing geometric morphometrics can improve accuracy in combination with other molecular methods for mosquito identification [100].

Mosquito species identification can also be further aided by molecular techniques based on differences in the sequences of different DNA genetic markers (e.g., COI, ITS2,  mitochondrial gene coding for , nuclear *white* gene, among others) [6, 7, 101]. PCR followed by sequencing of cytochrome c oxidase I (COI), the most commonly used genetic marker [102], has been employed to assess intraspecific variation in species such as *Aedes caspius*, a vector of chikungunya (CHIKV) and West Nile virus (WNV), in Iran [103], and to track the invasion of *Aedes japonicus japonicus* in Romania [104]. The COI is also used in the DNA barcode method for mosquito identification. A 648 base pair (bp) COI gene sequence is amplified from the genomic DNA of specimens and used against a barcoding database for comparison to other species [105]. This method successfully identified subgroups/complexes within *Culex* (*Culex*) from the Neotropics [106], the establishment of *Ae. scapularis* in the Florida peninsula [107], as well as studies on species composition and density of local mosquito populations in Asia and Europe [108–110]. Another example is using the ITS2 genetic marker. Differences in ITS2 sequences have led to elucidating different species complexes, such as identifying six cryptic species of the *Anopheles crucians* complex [111]. Information provided by these markers can be complementary and help further elucidate the relationships and species composition within complexes of cryptic species otherwise indistinguishable and even assess hidden diversity [6, 112–114].

Besides direct analysis of obtained DNA sequences, other assays based on molecular biology have been developed to differentiate species and assess mosquito diversity, among other uses. Enzyme-based methods such as restriction fragment length polymorphism (RFLP) and PCR–RFLP, based on the band size patterns emerging from the use of restriction endonucleases, can be reliable, even serving to differentiate between progenies, track gene inheritance, and segregate between populations, despite being labor-intensive [115–119]. Random amplified polymorphic DNA (RAPD) markers were developed for mosquitoes shortly after. These consist of the obtention of different patterns of bands in the electrophoresis of PCR fragments produced by short primers of arbitrary sequences; primer pair design drives the usefulness of this method, and it can range from aiding in species identification and assessing differences between mosquito populations [119–121].

Probe-based assays have also been developed to identify mosquito species. The TaqMan real-time quantitative (q)PCR uses fluorescent probes to detect specific DNA sequences during the extension of PCR products, and TaqMan assays have been developed to discriminate between mosquito species and identify members of cryptic species complexes, but it is limited to the use of single probes [116, 122]. Another probe-based approach is multiplex PCR, which solves the single-probe issue and uses multiple probes labeled with different fluorophores, which can further streamline mosquito identification with fewer limitations than the TaqMan qPCR assays [123]. Although these assays are highly reliable and provide accurate results, their significant cost may limit their accessibility and widespread use, particularly in resource-limited settings. This financial barrier can restrict their application to only certain regions or research settings, potentially hindering broader use in the most needed areas.

Notably, the recent development of modern algorithms and model-based digital tools could lead to relatively reliable automation of specimen identification using external morphology for regional species assemblies, where this could be a viable approach to support mosquito vector research and surveillance [124, 125].

##### The importance of capturing live mosquitoes and establishing colonies for research

Collecting and maintaining live mosquitoes can potentially support many aspects of mosquito vector research. Traditionally, obtaining progeny of species of difficult taxonomic identification could lead to circumventing potentially ambiguous morphologic characteristics in female specimens through the observation of confirming characteristics such as those observed in male mosquito genitalia and 4th instar larvae from progeny-derived specimens [106, 126]. Another critical aspect of maintaining a laboratory colony is that researchers are not limited to specific times of the year for mosquito collection to perform research and experimental work. With the availability of colonized species, researchers can secure enough samples to conduct experiments continuously.

Every mosquito collection method has its distinct application and limitations. For example, while the BG-Sentinel 2 trap remains the gold standard for *Aedes* surveillance, it can collect other insect species. CDC light trap-based collection offers a broader range of mosquito species. In addition, aspirators provide a practical approach to the indoor and outdoor collection of resting mosquitoes. At the same time, oviposition traps can help monitor the egg-laying of mosquito populations [75–77] and provide live biological material. Other collection techniques, such as HLC, can be highly efficient in representing the human-biting portion of mosquito species’ assemblages, but ethical concerns have pushed the need for developing, testing, and implementing safer collection methods. The use of subsets of these mosquito collection techniques varies and is determined by the biology of the species population under surveillance, the need for molecular testing of pathogens, the need to collect live specimens for colony establishment for different goals, and intrinsic needs driven by pathogen surveillance/detection.

In addition, simple redesigning approaches to widely used collection devices can prove useful in curbing the physical damage of samples to facilitate their identification or to extend their use for subsequent bioassays or the establishment of mosquito colonies. A great example of this approach is presented by the work carried out by Kim and collaborators, where they reconfigured existing traps such as CDC light traps, BG-Sentinel, and gravid traps to have mosquitoes aspirated toward containment without being forced to pass through a moving fan [81]. As illustrated in this work, redesigning can extend the potential uses of readily available traps and provide flexible trapping platforms for mosquito vector researchers.

#### Disease research/vector studies

Mosquito colonies are maintained in research settings to study their biology and ability to transmit pathogens. This allows researchers to understand disease transmission dynamics better and then develop and employ appropriate control strategies [127]. In maintaining stable populations in controlled environments, mosquito factors, such as genetic traits, behavior, and mosquito–pathogen interactions that affect their role as disease vectors, can be explored. If key features identifying the factors that contribute to a mosquito’s capacity to spread these diseases are found and studied in a laboratory setting, then they may be able to be translated into real-world applications.

Maintaining mosquito colonies is essential for investigating genetic resistance and developing new strategies to control mosquito populations. One example would be utilizing these colonies to develop new insecticide strategies to combat the increasing resistance displayed by those strains [128] or develop genetic control strategies to decrease disease transmission [129]. Data from these investigations are crucial for designing effective vector control methods and ensuring that insecticides remain viable to combat mosquito-borne diseases. In addition to surveying the viability of insecticides, exploring the genetic mechanisms that confer resistance to various control measures also ties into investigations such as this. Gaining better insight into how mosquitoes build resistance to pesticides is a fruitful endeavor. Maintained colonies provide a platform to test such endeavors and other alternative control strategies, adapting to the growing challenge of resistance and implementing up-to-date and sustainable solutions to combat the spread of mosquito-borne diseases. One point that has been debated for several years is the concern that mosquitoes maintained in colonies may no longer accurately reflect wild populations, as they often undergo inbreeding and are reared in insectary conditions that, while mimicking the environment, do not perfectly replicate it. However, despite this issue, having standardized mosquito colonies can significantly enhance the reproducibility of results across laboratories worldwide, facilitating meaningful comparisons of findings.

#### Genetic and behavioral studies

Laboratory colonies of vectors of disease can aid in understanding the complex interplay of factors that can potentially shape their competency as vectors and their epidemiological impact in real-life conditions by tying together environmental effects on both gene regulation and differences in their potential to transmit disease and be controlled through novel control techniques. For example, in *Ae. aegypti* exposed to ZIKV, gene expression and regulation changes associated with different temperature treatments have been observed [130] along with changes in this species’ capacity to transmit this disease with an optimal temperature of 29 °C (range of 22.7–34.7 °C).

Conducting behavioral research on mosquitoes is crucial to understanding key aspects of their biology, such as feeding preferences, mating patterns, host-seeking behaviors, and other factors that relate to their role as disease vectors [131]. As an example, it has been demonstrated that in *Ae. aegypti*, laboratory infection with DENV can affect the expression of genes involved in infection modulation in salivary glands and has the potential to alter blood-feeding behavior [132]. Moreover, colonies of *Anopheles gambiae* s.s. have helped in elucidating the role of the composition of bacterial communities on the human skin in mediating attraction [133], to name a few.

##### Pathogen identification

Pathogen identification in the invertebrate host primarily depends on molecular approaches (specifically viruses) or visual criteria (e.g., malaria parasite). Classical methods rely on in vitro DNA/RNA amplification, electron microscopy, and serum from infected hosts [134]. Although virus isolation is the standard method for identifying viruses, not all viruses can be cultured in the laboratory. Techniques such as PCR, RT–qPCR, and microarrays are reliable alternatives for identification. Degenerate RT–qPCR and microarrays, which rely on detecting highly conserved regions, have been widely used for viral detection in various mosquito species [135, 136]. These methods are specific, efficient, and highly sensitive for detecting and quantifying several viruses from field-collected mosquitoes [136–138]. Although these techniques offer several advantages, they depend on known viral genomes for viral discovery, limiting the exploration of natural viral diversity.

To circumvent the limitations of traditional methods in virus identification and enable a broader survey of the abundance and diversity of viruses, metagenomics is a practical approach for discovering new viruses and allowing investigations in different types of samples (reviewed in [139]). Metagenomics involves obtaining the DNA sequence from multiple organisms, typically from microbial communities where individual microbes cannot be isolated. Arboviruses account for more than 500 known species, with over 100 capable of infecting humans [140, 141]. These viruses make up most of the mosquito virome, along with insect-specific viruses [142]. Variations in metagenomics analyses applied in field-collected mosquitoes include vector-enabled metagenomics and RNA shotgun metagenomics [143–145]. In the former, viral particles are purified, DNA and/or RNA are extracted, and following an amplification step, the samples are sequenced. These metagenomic approaches have enabled the discovery of numerous novel viruses from different hosts in a single experiment, revealing the diversity of viruses from animals, plants, insects, and bacteria in the samples analyzed [143–145].

Using nondestructive techniques as an alternative method for detecting pathogens, aside from metagenomics, would be impactful because it not only identifies pathogens but also preserves the morphology of mosquitoes. Current gold-standard techniques for pathogen detection and vector incrimination in mosquitoes often cause damage to the body of the insect. For example, the analysis of malaria sporozoites in the salivary gland is conducted through microscopic dissection or PCR, both of which result in the destruction of the insect body and hinder its identification using standard taxonomical procedures [146–149].

Three main nondestructive techniques for pathogen detection, considering mosquito biology, are described: (1) Saliva analysis during blood feeding, which requires live mosquitoes and is typically applicable to lab-maintained specimens. While this technique can, in principle, be used with field-collected mosquitoes, it depends on the mosquito surviving long enough in laboratory conditions to initiate a blood meal, which is often challenging owing to stress, injury during capture, or environmental mismatch; (2) Feces analysis from refractory mosquitoes, which also requires live specimens and extended maintenance under controlled conditions to allow digestion and excretion. As with saliva analysis, applying this method to field-collected mosquitoes is limited by their potential inability to survive rearing in the lab; and (3) Near-infrared spectrometry (NIRS), which can be applied to both live and freshly killed mosquitoes, and is more flexible. NIRS can be performed on both lab-reared and field-collected specimens, although its accuracy may be affected by factors such as age, preservation status, or environmental variability in field samples [150]. Exploring these new nondestructive strategies could be helpful and beneficial, potentially contributing now or in the future to maintaining the essential requirements of taxonomic parasitological studies. This includes correctly identifying vector species and gathering data on the natural infection rate or the transmission of target pathogens. Therefore, exploring and utilizing nondestructive techniques to improve current strategies is crucial.

A limiting factor in this field is the lack of consensus or standardization on the best technique for preserving viruses in mosquitoes collected and transported to the laboratory for analysis. Some collections are performed in locations very far from where the samples will be analyzed, and it is unknown how much material is lost during this process. However, interesting experimental approaches have been carried out to test the potential of different preservation methods for viral RNA in mosquitoes, and readily available solutions have shown utility in room-temperature storage. For example, immersion in ethanol, propylene glycol, or commercial reagents such as DNA/RNA Shield has been tested and found effective in preserving viral RNA integrity. These preservation methods vary in cost and accessibility; ethanol and propylene glycol are inexpensive and widely available, making them practical choices for fieldwork, while DNA/RNA Shield offers high preservation quality but at a higher cost, which may limit its use in large-scale or resource-limited settings [151].

##### Pathogen infection in vector mosquitoes for host–pathogen interaction studies

Blood feeding is a fundamental vector behavior with epidemiological and biological consequences. It is through the blood meal that anautogenous species acquire the essential nutrients needed for egg production, but it is also how mosquitoes become infected with pathogens, or how transmission of the pathogens occurs [149]. Any pathogen ingested by the mosquito faces challenges or barriers that impact its replication or the progression of its cycle, which ultimately determines if, in a subsequent blood meal, it will infect another host. This ability to acquire, maintain, and transmit a pathogen is termed vector competence [152, 153]. The estimation of vector competence is crucial for comparing and understanding different epidemiological situations of vector-borne diseases. It is intimately correlated to vectorial capacity, defined by the potential of a vector to transmit a pathogen [153, 154].

The three most common methods used to infect mosquitoes for host–pathogen studies are: direct skin feeding (DSF), direct membrane feeding (DMF), and artificial membrane feeding (AMF). In DSF, laboratory-reared mosquitoes are placed directly on the skin of infected carriers, a method first developed by Muirhead-Thompson in the 1950s to measure malaria infection prevalence in an African village [155, 156]. This technique better mimics in vivo feeding conditions than any other method. As an alternative, DMF can be used when DSFs cannot be performed. In this method, freshly collected blood from infected carriers is placed in devices such as a double-jacketed glass cylinder [157] or the Hemotek [158] to keep the blood warm (human blood temperature) and artificially feed the mosquitoes. Comparison of both methods demonstrated similarity and can be used interchangeably depending on the experiments [159–161]. However, host attractiveness, immune responses, natural parasite densities, or natural host viremia cannot be replicated in direct membrane feeding. Another described method for infecting mosquitoes involves offering them two cotton stick ends fully soaked in infectious blood (Holicki et al., 2020; Heitmann et al., 2018). Alternatively, droplets of the infectious blood meal can be placed directly into a plastic vial, allowing mosquitoes to feed for 2 h (Heitmann et al., 2018). Both techniques eliminate the need for warming the blood, which simplifies the procedure and makes it more accessible, especially in settings with limited equipment. These approaches offer practical and effective alternatives to traditional feeding methods.

Frequently used in vectorial capacity studies, AMF presents as a substitute for mosquito infections whenever infected carriers or infected blood from carriers are not available. Obtaining ethical approval can be difficult in certain countries, and the recruitment of patients can be challenging, especially if the research is not being performed in places where the disease is present. In AMF, a vertebrate blood mixture with a cell-cultured virus or, in the case of malaria parasites, cultured gametocytes, is offered to mosquitoes to initiate the infection using the same devices as DMF to keep the blood warm. The ease of assessing different virus infections, the reproducibility of experiments, and the lack of animal use make AMF an attractive approach for mosquito infection studies. As an additional comment, it is worth mentioning that pathogen identification and artificial infection studies have been facilitated by the development and increased availability of novel mosquito cell lines from various species [162, 163].

Besides the obvious benefit of laboratory artificial infection of mosquitoes in vector incrimination [155, 156, 164–166], host–pathogen interaction studies through artificial infection have furthered our understanding of these complex relationships. For example, it has been observed that DENV (DENV-2 strain) can increase the time that *Ae. aegypti* females spend ingesting blood while decreasing their lifespan and having detrimental effects on their fitness [167]. Furthermore, artificial infection laboratory studies have helped in elucidating mechanisms such as collagen degradation of the basal lamina that could facilitate viral dissemination of CHIKV from the midgut in *Ae. aegypti* [168], or elucidate the vector competence differences in Zika virus (ZIKV) infection on standard laboratory colonies of *Ae. aegypti* [169]. Other artificial infection studies have shown that successive blood meals in *Aedes* mosquitoes significantly enhance the dissemination of viruses such as ZIKV, DENV, and CHIKV by facilitating their escape from the midgut, thus increasing transmission potential and explaining how *Ae. aegypti* manages to sustain explosive epidemics despite relatively low vector competence in single-feed infection trials [170]. Changes in blood-feeding can also be significant in shaping human–mosquito contact and altering disease transmission rates [171].

## Conclusions

With the availability of numerous tools and methodologies, the conclusion of this paper underscores the remarkable diversity of mosquito collection and identification options, as well as the extensive range of techniques for isolating and detecting pathogens. From classical trapping strategies and morphological identification to high-throughput sequencing and advanced molecular diagnostics, researchers now have an expansive toolkit to investigate vectors and the pathogens they transmit. Integrating these methodologies into infection studies is critical for understanding the complexities of host–pathogen interactions, offering fundamental insights for designing more targeted and effective vector control strategies. Yet, the responsibility of selecting, combining, and applying these methods with scientific rigor rests entirely on the researcher. A carefully tailored study design, anchored in a critical review of proven approaches and aligned with specific research goals, is essential for ensuring robust, reproducible results and meaningful contributions to the field of vector biology. Scientific capabilities and technologies have progressed significantly since the initial description of most known vector-borne pathogens, as reviewed throughout this paper. Today, it is possible to apply molecular biology and both in vivo and in vitro techniques to detect and characterize novel pathogens, assess their potential as human disease agents, and explore their transmission dynamics. In parallel, advances in artificial intelligence and machine learning promise to revolutionize mosquito collection and identification, with the potential to automate and scale processes in ways that could be transformative for field and lab work. Nonetheless, we emphasize the enduring value of traditional methods and the critical role of experienced taxonomists whose expertise can resolve complex identifications and fill in the gaps where machines still fall short. As we prepare for future threats and aim to forecast emerging epidemics, a balanced approach that embraces technological innovation and foundational knowledge will be key to protecting public health.

## Supplementary Information

**Additional file 1: Fig. S1.** Different methods for collecting mosquitoes. **A)** BG-Sentinel 2 trap with BG-Lure (Photo: M. Futo); **B)** CO_2_-baited CDC miniature light trap (Ph. S. Duran); **C)** Small-diameter mosquito aspirator (Photo: S. Kroening); **D)** Ovicup made from small painted coffee cans with balsa wood pieces as egg-laying substrate (Photo: S. Duran). Photos provided by authors and FMEL members.

## Data Availability

Data supporting the main conclusions of this study are included in the manuscript.
