# Supplementary figures and images for: The toolbox for mosquito vector research
Source: Parasit Vectors. 2025 Sep 24;18:389. doi: 10.1186/s13071-025-07008-2 (PMC12462310; doi:10.1186/s13071-025-07008-2)

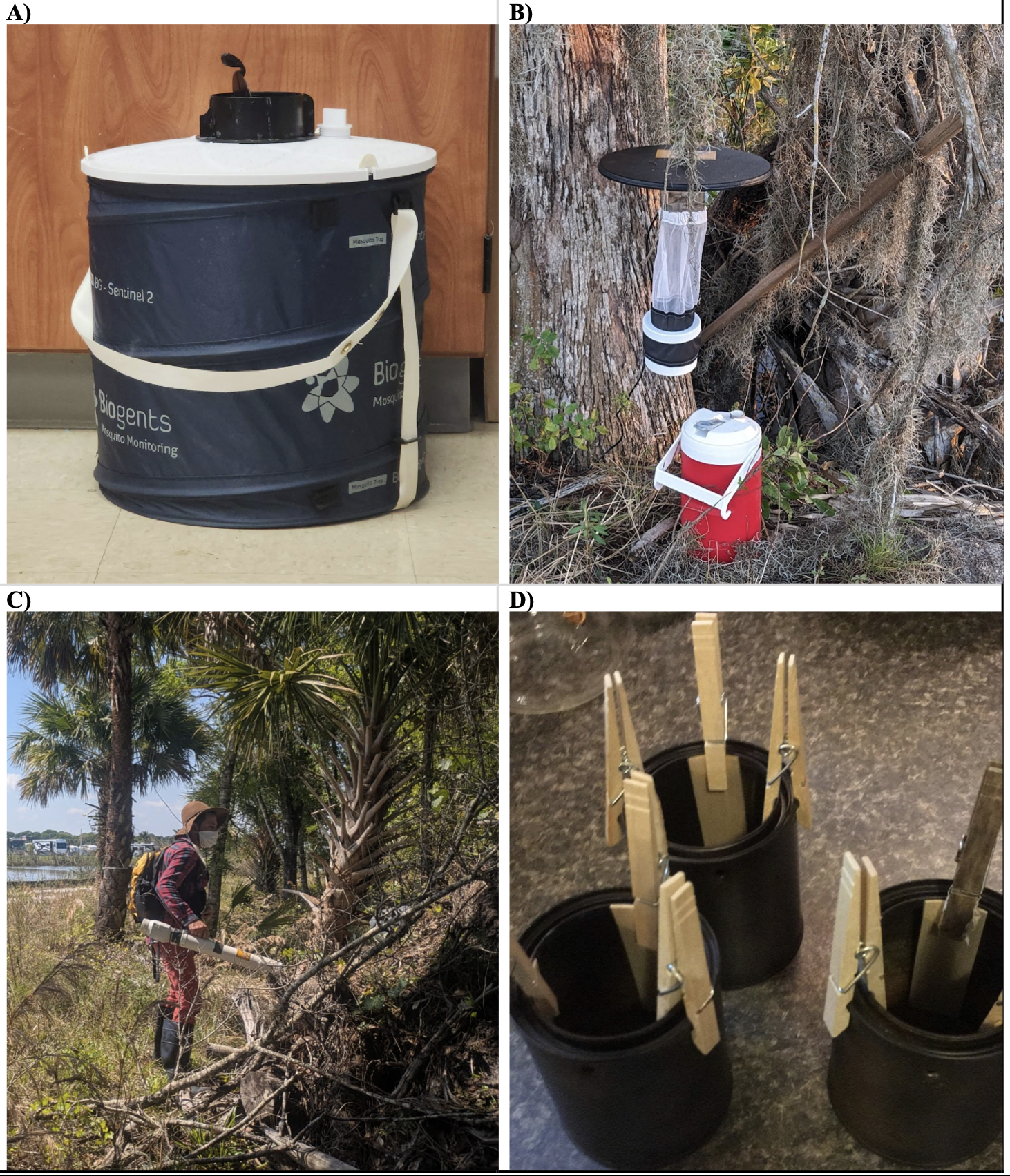

Supplement: Supplementary file 1 — Additional file 1: Fig. S1. Different methods for collecting mosquitoes. A) BG-Sentinel 2 trap with BG-Lure (Photo: M. Futo); B) CO2-baited CDC miniature light trap (Ph. S. Duran); C) Small-diameter mosquito aspirator (Photo: S. Kroening); D) Ovicup made from small painted coffee cans with balsa wood pieces as egg-laying substrate (Photo: S. Duran). Photos provided by authors and FMEL members. [file 13071_2025_7008_MOESM1_ESM.png]
